# Circulating RNA as a Functional Component of Liquid Biopsy in Cancer: Concepts, Classification, and Clinical Applications

**DOI:** 10.3390/ijms27052403

**Published:** 2026-03-05

**Authors:** Kyung-Hee Kim, Byong Chul Yoo

**Affiliations:** 1 Department of Applied Chemistry, School of Science and Technology, Kookmin University, Seoul 02707, Republic of Korea; kyungheekim@kookmin.ac.kr; 2 Antibody Research Institute, Kookmin University, Seoul 02707, Republic of Korea; 3 Diagnostic Research Team, InnoBation Bio R&D Center, Seoul 03929, Republic of Korea

**Keywords:** circulating RNA, liquid biopsy, liquid transcriptome, functional biomarkers, early cancer detection

## Abstract

Liquid biopsy has become an integral component of precision oncology, with circulating tumor DNA serving as the dominant analyte for genomic profiling and disease monitoring. However, DNA-based approaches are intrinsically limited in their ability to capture dynamic cellular states, functional adaptation, and tumor–host interactions. Circulating RNA has emerged as a complementary class of liquid biopsy biomarkers that reflects active transcriptional programs and systemic biological responses. In this review, we conceptualize circulating RNA as a liquid transcriptome and propose a structured classification framework based on physical carriers, RNA biotypes, and layers of biological interpretation. We describe how circulating RNA signals encode tissue-of-origin information, cell-state dynamics, and host immune responses, thereby enabling system-level insight into cancer biology beyond mutation-centric analyses. Recent large-scale profiling efforts and advances in extracellular RNA characterization further support the biological relevance and analytical feasibility of circulating RNA across diverse biofluids. We discuss emerging applications of circulating RNA across the cancer continuum, including early cancer detection and multi-cancer screening, tissue-of-origin inference, longitudinal monitoring of treatment response, detection of adaptive resistance, and immunotherapy stratification. In parallel, we critically examine key technical, analytical, and computational challenges that currently limit reproducibility and clinical translation, emphasizing the importance of standardized workflows, transparent reporting, and multi-center validation. Finally, we outline future directions for integrating circulating RNA with genomic and proteomic biomarkers, supported by advances in artificial intelligence and machine learning. Collectively, this review positions circulating RNA as a functionally informative and clinically promising component of next-generation liquid biopsy strategies in oncology.

## 1. Introduction

Liquid biopsy has reshaped the landscape of cancer diagnostics by enabling minimally invasive access to tumor-associated molecular information through peripheral blood sampling [1,2,3,4]. Over the past decade, circulating tumor DNA (ctDNA) has dominated the field, demonstrating clinical utility in mutation profiling, detection of minimal residual disease, monitoring of clonal evolution, and assessment of therapeutic resistance [5,6,7,8]. These advances have firmly established ctDNA as a cornerstone of precision oncology [1,9].

Nevertheless, ctDNA-based approaches are intrinsically constrained by the nature of the information they encode [7,10]. As fragments of genomic DNA released primarily through cell death, ctDNA reflects the structural presence of tumor-derived genetic alterations but provides limited insight into dynamic biological processes such as transcriptional activity, cellular stress responses, immune interactions, and adaptive reprogramming under therapeutic pressure [6,9]. Consequently, ctDNA often lags behind functional changes in tumor behavior and host response, particularly in early disease stages or during treatment adaptation [5,11].

Circulating RNA represents a complementary and conceptually distinct class of liquid biopsy biomarkers [12,13]. By capturing transcriptional outputs rather than static genomic blueprints, circulating RNA conveys real-time information on cellular identity, functional state, and biological activity of both tumor and host tissues [14,15]. RNA molecules respond rapidly to environmental cues, metabolic stress, and therapeutic perturbations, positioning circulating RNA as a functional liquid biopsy capable of interrogating not only the presence of cancer but also its biological behavior and systemic impact [13,16].

Importantly, circulating RNA is not a monolithic entity [17,18,19]. RNA molecules circulate in multiple physical forms, including freely circulating cell-free RNA (cfRNA), RNA encapsulated within extracellular vesicles (EVs), and RNA associated with ribonucleoprotein complexes [20,21,22,23]. In addition, circulating RNA encompasses diverse biotypes—such as messenger RNA (mRNA), microRNA (miRNA), long non-coding RNA (lncRNA), and circular RNA (circRNA)—each contributing distinct layers of regulatory and diagnostic information [24,25,26]. Advances in low-input RNA sequencing, improved library preparation protocols, and computational deconvolution methods have substantially enhanced the feasibility of analyzing these heterogeneous RNA populations [27,28,29,30,31,32,33].

In this review, we present a comprehensive conceptual framework for circulating RNA as a liquid transcriptome, propose a structured classification system based on physical carriers, RNA biotypes, and biological meaning, and critically examine emerging applications in cancer diagnosis and therapy. We further discuss technical challenges and outline future directions for clinical translation, with a particular focus on integrating circulating RNA into functional precision oncology [34].

In this context, the term *functional liquid biopsy* refers to biomarker strategies that extend beyond mutation-centric detection of tumor-derived DNA. Whereas traditional liquid biopsy has largely focused on identifying genomic alterations to infer tumor presence and clonal architecture [1,7], a functional approach emphasizes dynamic biological states, including transcriptional activity, cellular stress responses, and tumor–host interactions [13,15]. Circulating RNA therefore represents not a replacement for DNA-based assays, but a complementary modality that captures temporally responsive and system-level biological information not directly encoded in the genome [9,13].

## 2. Conceptual Framework of Circulating RNA as a Liquid Transcriptome

Circulating RNA can be broadly defined as RNA molecules present in the circulation that originate from tumor cells, normal tissues, immune cells, and stromal compartments [15,17]. Collectively, these molecules constitute the liquid transcriptome, representing an integrated snapshot of transcriptional activity across the organism at a given time point [12,14,35,36].

A central conceptual distinction between circulating RNA and circulating DNA lies in the type of biological information conveyed [9,13]. ctDNA reflects genomic alterations accumulated over time and released primarily through apoptosis or necrosis [5,7]. In contrast, circulating RNA reflects active transcriptional programs, enabling interrogation of cellular function, state transitions, and biological responsiveness [12,15]. This distinction has important implications for temporal resolution: RNA signals often change on the scale of hours to days, whereas DNA-based signals may remain stable despite substantial functional reprogramming [1,6,13].

**Figure 1:**
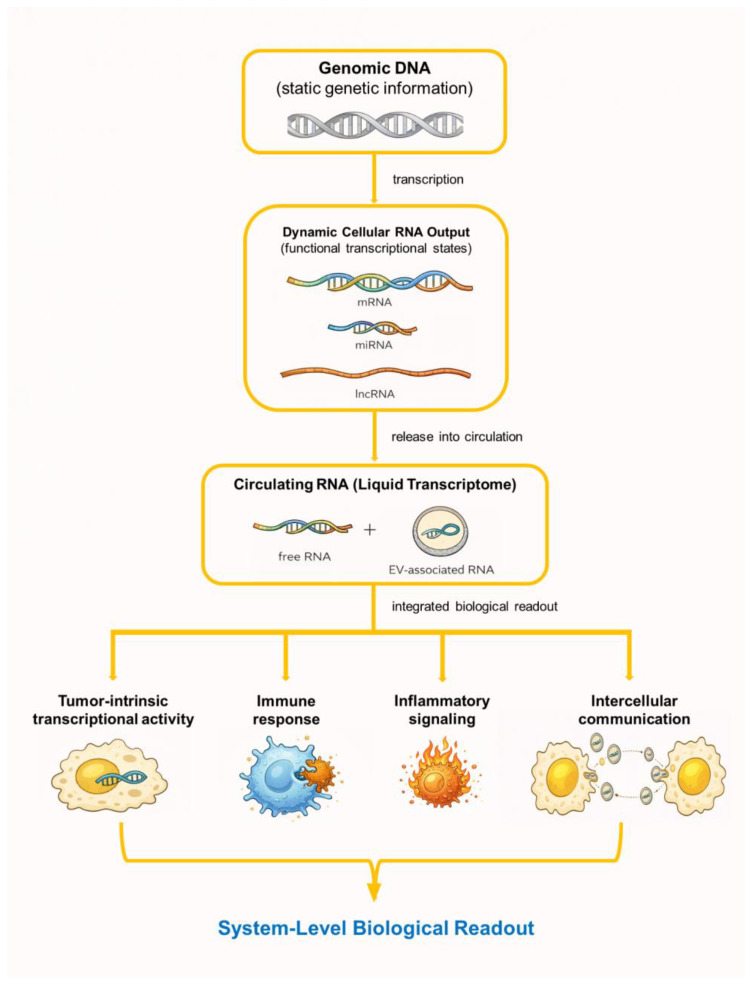
Conceptual framework of circulating RNA as a liquid transcriptome. This schematic illustrates circulating RNA as an integrated and dynamic transcriptomic readout that reflects tumor-intrinsic transcriptional activity, host immune and inflammatory states, and intercellular communication mediated in part by extracellular vesicles. In contrast to static genomic information derived from circulating DNA, circulating RNA captures functional and context-dependent biological states. Figures were assembled and edited by the authors using Microsoft PowerPoint, incorporating graphical elements for schematic illustration.

Another defining feature of circulating RNA is its systemic nature [13,14,37]. While tumor-derived transcripts contribute to the circulating RNA pool, host-derived RNA—particularly from immune and inflammatory cells—often dominates the signal [15,35]. Rather than representing a limitation, this characteristic enables circulating RNA to capture tumor–host interactions, immune activation states, and systemic responses to malignancy or therapy [9,15,38]. Accordingly, interpretation of circulating RNA requires a system-level perspective that extends beyond tumor-centric paradigms [9,13]. A schematic overview of circulating RNA as an integrated liquid transcriptome is presented in Figure 1.

## 3. Physical Origins and Carriers of Circulating RNA

Circulating RNA originates from multiple biological processes that collectively shape its composition in blood. Passive release through apoptosis or necrosis contributes fragmented RNA species derived from dying tumor and normal cells [5,12]. In parallel, active secretion mechanisms—including selective RNA packaging into extracellular vesicles—reflect biologically regulated intercellular communication pathways [20,22]. Platelets and immune cells can also acquire and redistribute RNA signals, further contributing to the circulating transcriptome and amplifying tumor-associated signatures [19,39]. These diverse origins underscore that circulating RNA represents not merely cellular debris, but a composite signal integrating tumor-intrinsic activity and systemic host responses [15,36].

### 3.1. Cell-Free RNA

Cell-free RNA (cfRNA) refers to RNA fragments freely circulating in plasma or serum [12,17]. cfRNA is typically highly fragmented and present at low abundance, reflecting rapid turnover and susceptibility to degradation by ribonucleases [15,40]. Despite these challenges, cfRNA retains tissue- and cell-type-specific expression patterns that can be exploited for tissue-of-origin inference and disease detection [12,14]. The transient nature of cfRNA confers both advantages and limitations [15]. On one hand, cfRNA is well suited for capturing acute biological changes, such as rapid transcriptional responses to therapy or stress [13]. On the other hand, cfRNA analysis is highly sensitive to pre-analytical variables, including sample collection, processing time, and storage conditions [40,41,42,43]. Standardization of these factors remains critical for reproducible cfRNA profiling [9,41].

### 3.2. Extracellular Vesicle-Associated RNA

Extracellular vesicles, including exosomes and microvesicles, encapsulate RNA molecules within lipid bilayers, providing protection from enzymatic degradation [20,22,44]. EV-associated RNA is often selectively packaged, suggesting active biological processes rather than passive release [20,22,44,45,46]. This selectivity enhances biological interpretability and stability, making EV-associated RNA an attractive target for diagnostic and prognostic applications [18,34,47,48,49,50]. In cancer, EV-derived RNA has been implicated in intercellular communication, tumor progression, immune modulation, and pre-metastatic niche formation [22,51,52]. These functional roles further support the relevance of EV-associated RNA as a biologically meaningful circulating biomarker [18,36].

### 3.3. Ribonucleoprotein-Associated RNA

A subset of circulating RNA is bound to ribonucleoprotein complexes, such as Argonaute proteins or lipoproteins [21,23]. This carrier form is particularly relevant for miRNAs, which frequently circulate in protein-bound states [21]. Ribonucleoprotein association contributes to RNA stability and influences detection strategies, particularly for small RNA sequencing or targeted assays [15,17].

### 3.4. Conceptual Integration of Carrier-Dependent Properties

While the physical carriers of circulating RNA—cell-free RNA, extracellular vesicle–associated RNA, and ribonucleoprotein-bound RNA—determine molecular stability, protection from degradation, and analytical accessibility, these categories alone do not define the clinical or biological relevance of circulating RNA signals [39]. Rather, carrier-dependent properties should be viewed as enabling layers that shape how transcriptional information is preserved and detected in the circulation.

Similarly, classification by RNA biotype provides a molecular vocabulary through which circulating transcriptomic signals are expressed, but does not fully capture their functional or clinical significance. The translational value of circulating RNA ultimately resides not in its physical form or molecular class, but in the biological meaning encoded by its expression patterns.

Accordingly, interpretation of circulating RNA requires a hierarchical framework in which physical carriers represent the mode of signal transport, RNA biotypes define the molecular constituents, and biological interpretation constitutes the central layer linking circulating RNA to cancer diagnosis and treatment. This perspective provides the conceptual foundation for understanding how diverse circulating RNA species converge into clinically actionable signals, as discussed in the following section. The carrier-dependent properties of circulating RNA are summarized in Table 1.

## 4. RNA Biotype-Based Classification

Circulating RNA encompasses multiple RNA biotypes with distinct biological roles and analytical characteristics [13,24,25]. mRNA fragments retain tissue-specific expression patterns and enable inference of transcriptional programs active in tumors or host tissues [12,14,53]. These features make circulating mRNA a powerful tool for tissue-of-origin analysis and functional state assessment [14,15].

miRNAs represent the most extensively studied circulating RNA species due to their stability, abundance, and regulatory roles in oncogenesis [17,18,24]. Numerous miRNAs have been implicated in cancer development, progression, and therapeutic resistance [11,18,54], although single-miRNA biomarkers often lack sufficient specificity for clinical application [9,18].

lncRNAs exhibit greater tissue and disease specificity but pose analytical challenges related to lower abundance and incomplete annotation [24,25]. circRNAs, characterized by covalently closed loop structures, demonstrate exceptional stability and are emerging as potential cancer biomarkers [17,18,26,55], although their functional interpretation remains an active area of research [15,26].

Integration of multiple RNA biotypes can enhance diagnostic robustness by capturing complementary regulatory layers within the circulating transcriptome. The major circulating RNA biotypes and their analytical features are summarized in Table 2.

## 5. Biological Meaning of Circulating RNA Signals

From a functional perspective, circulating RNA signals can be categorized according to the biological information they convey [9,13,15]. Tissue-of-origin signatures arise from expression of organ-specific transcripts and enable localization of disease processes [12,14]. Cell-state-associated signals reflect proliferation, hypoxia, metabolic stress, epithelial–mesenchymal transition, and other adaptive programs relevant to cancer progression [15].

Host-response signals, including immune activation and inflammatory pathways, provide insight into tumor–host interactions and therapeutic responsiveness [6,15,38]. These signals are particularly relevant in the context of immuno-oncology, where systemic immune states critically influence treatment outcomes [9,38].

Distinguishing functional biomarkers from correlative signals remains a central challenge in circulating RNA research and underscores the need for integrative analytical frameworks [9,13,16]. These layers of biological interpretation and their clinical relevance are summarized in Table 3, which provides a conceptual bridge to the diagnostic and therapeutic applications discussed in the following sections.

## 6. Circulating RNA in Cancer Diagnosis

### 6.1. Early Cancer Detection and Screening

Early cancer detection remains one of the most compelling yet challenging goals of liquid biopsy research [1,5,16]. In contrast to ctDNA-based approaches, which rely on the presence of tumor-derived genomic material and therefore often suffer from limited sensitivity in early-stage disease, circulating RNA offers access to transcriptional signals that may arise from small tumor burdens or even premalignant states [5,12,13]. Circulating RNA signatures can capture both tumor-intrinsic transcriptional activity and host responses, such as immune activation or inflammatory remodeling, that accompany early oncogenesis [15,38]. This dual contribution enables detection of biologically active disease even when tumor-derived DNA fragments are scarce [12,14].

Several studies have demonstrated that cfRNA or EV-associated RNA profiles can distinguish early-stage cancers from healthy controls with performance comparable to or exceeding that of mutation-based assays [15,16,29,35,56].

However, the increased sensitivity of circulating RNA comes at the cost of greater complexity [9,13]. Unlike ctDNA mutations, which provide relatively binary signals, transcriptomic signatures are inherently continuous and context-dependent [6,15]. As a result, early detection strategies based on circulating RNA often rely on multigene signatures and machine learning-based classifiers rather than individual biomarkers [57].

Beyond simple signal abundance, several biological and technical factors contribute to the apparent sensitivity advantage of circulating RNA-based assays in early-stage disease. In the earliest phases of tumor development, the absolute fraction of tumor-derived DNA in circulation is often exceedingly low, limiting the detectability of somatic mutations by ctDNA-based approaches [5,7,12,58]. In contrast, transcriptional signals captured through circulating RNA reflect not only tumor-intrinsic gene expression but also amplified downstream responses from the tumor microenvironment and host tissues, including stromal remodeling, immune activation, and inflammatory signaling [12,14,15]. This transcriptional amplification effect enables circulating RNA profiles to register disease-associated perturbations even when direct tumor burden is minimal [14,15,29].

Importantly, early oncogenic processes frequently manifest as coordinated changes in gene expression programs rather than as stable, clonally fixed genetic alterations. Such transcriptional reprogramming can precede overt tumor expansion, shedding, or radiographic detectability, thereby providing a temporal window in which circulating RNA signatures may outperform mutation-centric assays [9,13,15]. Furthermore, because circulating RNA integrates signals from multiple cellular sources, including immune and non-malignant compartments, it may capture systemic responses to nascent malignancy that are inherently invisible to tumor-restricted DNA-based analyses [12,15,35]. Together, these features position circulating RNA not merely as an alternative analyte, but as a functionally distinct biomarker modality for early cancer detection and multi-cancer screening [15,16,29,35].

### 6.2. Multi-Cancer Detection and Tissue-of-Origin Inference

Multi-cancer early detection represents a particularly attractive application of circulating RNA [9,51,59,60]. Because RNA expression patterns retain tissue specificity, circulating RNA enables inference of tissue-of-origin through organ-enriched transcriptomic signatures [12,14,61]. This capability addresses a key limitation of many multi-cancer ctDNA assays, which may detect malignancy but provide limited information regarding tumor localization [1,55]. Transcriptome-based tissue-of-origin inference leverages expression patterns from both protein-coding and non-coding RNAs, often integrating signals across multiple carriers and RNA biotypes [15,17].

Importantly, host-derived signals can further refine tissue localization by reflecting organ-specific immune or stromal responses [15,38]. Such integrative approaches position circulating RNA as a promising platform for biologically informed multi-cancer screening [9,13].

### 6.3. Discrimination of Malignant and Benign Conditions

A persistent challenge in cancer screening is the differentiation of malignant disease from benign or inflammatory conditions [9]. Circulating RNA offers potential advantages in this context by capturing functional differences in transcriptional programs rather than relying solely on the presence of genetic alterations [13,15,62]. For example, transcriptional signatures associated with sustained proliferative signaling, metabolic reprogramming, or immune evasion may distinguish malignant processes from transient inflammatory responses [36].

Nevertheless, overlap between cancer-associated and inflammation-associated transcriptomic signals remains a significant confounder, underscoring the need for careful feature selection and validation in diverse clinical cohorts [9,57,63].

## 7. Circulating RNA in Cancer Treatment

### 7.1. Monitoring Treatment Response

In the therapeutic setting, circulating RNA provides a dynamic window into treatment response [13,15]. Transcriptional changes often precede detectable changes in tumor size or genomic composition, enabling earlier assessment of therapeutic efficacy [6,9]. Longitudinal monitoring of circulating RNA can reveal rapid suppression or reactivation of oncogenic pathways, offering actionable insights into treatment dynamics [13,16,64].

Unlike ctDNA, which primarily reflects changes in tumor burden or clonal composition, circulating RNA captures functional adaptation at the cellular and systemic levels [5,7,13,15,65]. This distinction is particularly relevant for therapies that induce transcriptional reprogramming without immediate tumor cell death [6,9,66].

### 7.2. Detection of Adaptive Resistance

Adaptive resistance to cancer therapy frequently involves transcriptional rewiring rather than acquisition of new genetic mutations [6,67]. Circulating RNA is uniquely suited to capture such adaptations, including activation of compensatory signaling pathways, stress response programs, and phenotypic plasticity [13,15].

For example, emergence of epithelial–mesenchymal transition signatures or metabolic reprogramming patterns in circulating RNA may signal impending therapeutic failure [16]. Early detection of these changes could enable timely modification of treatment strategies before overt clinical progression [9,13].

### 7.3. Applications in Immuno-Oncology

Immunotherapy has highlighted the importance of host immune states in determining treatment outcomes [9,67]. Circulating RNA enables non-invasive profiling of immune activation, exhaustion, and cytokine signaling, providing insights that are difficult to obtain from tumor-derived DNA alone [15,35,38].

Transcriptomic signatures reflecting interferon signaling, T cell activation, or immunosuppressive pathways have been associated with response or resistance to immune checkpoint inhibitors [11,38,68]. By integrating tumor-intrinsic and immune-derived RNA signals, circulating RNA offers a holistic approach to monitoring immunotherapeutic efficacy [9,13].

In the context of immuno-oncology, the utility of circulating RNA extends beyond tumor burden estimation to the functional inference of host immune states. Immune checkpoint blockade and other immunotherapies exert their effects primarily by modulating pre-existing immune activity rather than directly targeting tumor cells, rendering static genomic alterations insufficient to capture treatment-relevant biology [9,38]. Circulating RNA profiles, by contrast, can reflect dynamic transcriptional programs associated with immune activation, exhaustion, and immune evasion, including interferon signaling, cytotoxic T-cell activity, and inflammatory pathways [15,35,38].

Importantly, such immune-related transcriptional states are often systemic and temporally variable, evolving in response to therapy and tumor–host interactions [69]. Longitudinal analysis of circulating RNA therefore offers a means to monitor immune engagement and functional response trajectories over time, potentially preceding radiographic changes or clinical response assessments [9,15,70]. Because these signals may arise from multiple immune and non-malignant compartments, circulating RNA enables a systems-level view of immunotherapy response that is inherently difficult to achieve through tumor-restricted or mutation-centric assays alone [12,15,35]. Collectively, these features position circulating RNA as a promising modality for immunotherapy guidance, response monitoring, and the early detection of immune escape or functional exhaustion [9,15,38].

### 7.4. Mini-Synthesis of Diagnostic and Therapeutic Applications

Taken together, applications of circulating RNA in cancer diagnosis and treatment underscore a fundamental distinction from mutation-centric liquid biopsy approaches. Whereas circulating DNA primarily reflects tumor burden and clonal architecture, circulating RNA captures functional adaptation, biological state transitions, and tumor–host interactions across the disease continuum. In diagnostic settings, this enables detection of biologically active malignancy even under conditions of low tumor burden, while preserving tissue-of-origin information. In therapeutic contexts, circulating RNA provides early insight into transcriptional reprogramming, adaptive resistance, and immune dynamics that often precede radiographic or genomic evidence of progression.

**Figure 2:**
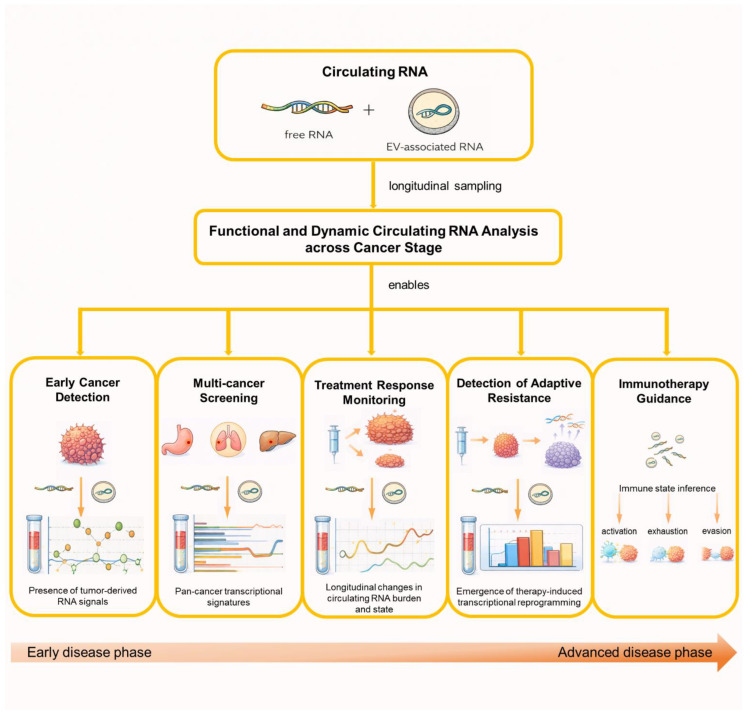
Applications of circulating RNA across the cancer continuum. Circulating RNA enables longitudinal and functional molecular assessment across the cancer continuum, from early cancer detection and multi-cancer screening to treatment response monitoring, detection of adaptive resistance, and immunotherapy guidance. Repeated profiling of circulating RNA supports dynamic inference of tumor and immune states over time. Figures were assembled and edited by the authors using Microsoft PowerPoint, incorporating graphical elements for schematic illustration.

These features position circulating RNA not merely as an alternative analyte, but as a functionally complementary layer of liquid biopsy, capable of informing clinical decision-making at stages where genomic signals alone may be insufficient. The challenge, therefore, is not whether circulating RNA is biologically informative, but how its complexity can be harnessed in a robust, reproducible, and clinically actionable manner. An overview of circulating RNA applications across the cancer continuum is illustrated in Figure 2.

## 8. Technical and Analytical Challenges

Beyond assay-specific considerations, the clinical translation of circulating RNA biomarkers is further constrained by challenges related to standardization, reproducibility, and cross-cohort comparability. Pre-analytical variables, including blood collection, processing time, RNA stabilization, and storage conditions, can introduce substantial variability in circulating RNA profiles, complicating inter-study comparisons [17,40,41,42,71]. At the analytical level, differences in library preparation, sequencing depth, and bioinformatic pipelines may amplify batch effects or platform-dependent biases, particularly for low-abundance transcripts [27,28,72].

These sources of technical heterogeneity underscore the need for harmonized workflows, rigorous quality control, and multi-center validation to ensure that circulating RNA signatures identified in discovery cohorts remain robust under real-world clinical conditions [9,13,73,74,75,76]. Without such standardization, reproducible yet fragile transcriptomic signals may fail to generalize beyond their original study context, limiting clinical utility despite promising initial performance.

### 8.1. Pre-Analytical Variability

Pre-analytical factors represent one of the most significant sources of variability in circulating RNA analysis [17,40]. Differences in blood collection tubes, processing times, centrifugation protocols, and storage conditions can profoundly influence RNA yield and composition [40,41,42]. Hemolysis and residual cellular contamination further complicate interpretation by introducing confounding transcripts [17,40].

Standardization of pre-analytical workflows is therefore essential for reproducibility and cross-study comparability, particularly in multi-center clinical trials [9,41].

### 8.2. Analytical Limitations

Analytical challenges include low RNA input quantities, fragmentation, and uneven transcript coverage [15,17]. Library preparation methods can introduce bias, particularly for low-abundance transcripts or specific RNA biotypes [27,28]. Batch effects and platform-dependent variability further complicate quantitative comparisons across datasets [15,17].

Addressing these limitations requires careful experimental design, inclusion of appropriate controls, and adoption of robust normalization strategies [9,27,30].

### 8.3. Computational Challenges

Computational analysis of circulating RNA data poses unique challenges due to high dimensionality, low signal-to-noise ratios, and mixed biological origins [13,15]. Source deconvolution—distinguishing tumor-derived from host-derived signals—remains a critical and unresolved problem [13,15].

Machine learning approaches offer powerful tools for feature selection and classification but carry risks of overfitting and limited generalizability [13,57,73]. Transparent modeling strategies and external validation are essential to ensure clinical robustness [9,57].

## 9. Clinical Translation and Validation

### 9.1. Study Design Considerations

The clinical translation of circulating RNA biomarkers requires rigorous and carefully structured study designs, including the use of appropriate control populations, longitudinal sampling strategies, and clinically meaningful endpoints [1,9,16]. As with other liquid biopsy modalities, retrospective analyses can provide valuable biological insights and generate testable hypotheses; however, they are insufficient to establish clinical utility on their own. Prospective validation in well-defined patient cohorts remains essential to demonstrate robustness, reproducibility, and real-world applicability [9,11,77].

In the context of circulating RNA, study design considerations are further complicated by biological and technical heterogeneity, including variability in RNA carriers, RNA biotypes, and analytical platforms. These factors necessitate harmonized pre-analytical and analytical workflows to ensure that observed signals reflect true biological variation rather than technical artifacts. Without such rigor, promising circulating RNA signatures risk failing during external validation or clinical implementation.

### 9.2. Clinical Utility Versus Statistical Performance

High analytical sensitivity and specificity, while important, are not sufficient criteria for clinical adoption of liquid biopsy assays [1,9]. Circulating RNA-based tests must ultimately demonstrate that they provide actionable information that improves clinical decision-making, patient outcomes, or healthcare efficiency beyond existing diagnostic modalities [9,10,13]. This distinction between statistical performance and clinical utility is particularly critical in screening and disease monitoring settings, where false positives, overdiagnosis, or unclear clinical interpretation can lead to unintended harm [6,9,11].

Compared with DNA-based assays, circulating RNA approaches are uniquely positioned to capture dynamic biological states, including transcriptional activity, immune responses, and treatment-induced adaptations. However, the clinical value of such information depends on whether these signals can be reliably interpreted and translated into defined clinical actions. As a result, circulating RNA biomarkers must be evaluated not only on their ability to discriminate disease states, but also on their capacity to inform clinically relevant decisions within specific therapeutic contexts.

In practical terms, the clinical value of circulating RNA will depend on whether it can alter management decisions earlier than existing modalities. For example, the emergence of transcriptional signatures associated with adaptive resistance may prompt modification of targeted therapy before radiographic progression becomes evident [6,67]. In immunotherapy settings, immune-related RNA signatures—such as interferon-γ-associated programs—may help distinguish true progression from pseudo-progression, thereby informing decisions to continue or discontinue therapy [38,68]. Similarly, sustained suppression of oncogenic transcriptional programs in circulation could support treatment de-escalation strategies in selected patients. Such decision-oriented applications represent the critical threshold for translating transcriptomic biomarkers from statistical discrimination to actionable clinical utility [9,13].

### 9.3. Regulatory and Implementation Perspectives

Regulatory approval of circulating RNA-based diagnostics will require clear demonstration of analytical validity, clinical validity, and clinical utility, consistent with established frameworks for molecular diagnostic assays [1,9]. Experiences from the clinical translation of ctDNA assays underscore the importance of standardized protocols, robust quality control measures, and clearly defined clinical indications [1,7,41]. In the absence of such standardization, variability in sample handling, RNA extraction, library preparation, and data analysis continues to undermine reproducibility across studies and institutions.

Despite these challenges, a growing body of clinical and translational evidence supports the feasibility of circulating RNA profiling across diverse cancer types and clinical scenarios [78,79]. Studies have reported potential utility in early cancer detection, disease characterization, treatment response monitoring, and immunotherapy stratification, particularly in settings where functional and dynamic biological information is clinically relevant. Representative examples of these clinical and translational studies are summarized in Table 4, highlighting both the promise and the current limitations of circulating RNA-based approaches in oncology. While multiple meta-analyses have evaluated specific RNA classes—particularly circulating miRNAs—in individual cancer types [17,18], the studies summarized here emphasize transcriptome-level and integrative approaches that illustrate broader functional applications of circulating RNA.

Collectively, these findings indicate that while circulating RNA biomarkers have not yet achieved routine clinical implementation, they offer complementary and functionally informative signals that extend beyond static genomic alterations. Continued progress will depend on systematic prospective validation, improved standardization, and careful alignment of circulating RNA assays with clearly defined clinical questions.

## 10. Future Perspectives

Despite increasing technical sophistication and growing biological insight, no single circulating biomarker modality has yet proven sufficient to address the full spectrum of clinical questions encountered across the cancer continuum. DNA-based assays excel at capturing tumor-derived genomic alterations and disease burden, but provide limited information on dynamic cellular states, transcriptional adaptation, and host–tumor interactions. Conversely, circulating RNA offers a functional and temporally responsive readout of biological activity, yet remains vulnerable to pre-analytical variability and context-dependent interpretation. These complementary strengths and limitations underscore the need for integrated liquid biopsy strategies rather than continued reliance on any single analyte class.

From a clinical perspective, integration is particularly relevant in scenarios where static genomic information alone fails to fully explain therapeutic response, resistance, or toxicity. Combining circulating RNA with genomic, proteomic, or clinical data may enable more robust biological inference, reduce false-positive signals, and improve interpretability by anchoring transcriptional changes to underlying molecular or phenotypic contexts. Importantly, integration should not be viewed as a simple aggregation of multiple biomarkers, but as a hypothesis-driven framework in which each modality addresses a distinct layer of disease biology. Such an approach has the potential to enhance clinical relevance while mitigating the limitations that currently constrain the standalone use of circulating RNA assays.

### 10.1. Integration with Multi-Modal Liquid Biopsy

Future liquid biopsy strategies are increasingly expected to adopt multi-modal designs that integrate circulating RNA with other analytes, including circulating tumor DNA, protein biomarkers, and clinical variables [9,13,81,82,83]. Such integrative approaches reflect the growing recognition that distinct biomarker classes capture complementary dimensions of cancer biology. While ctDNA provides high specificity for tumor-derived genomic alterations and disease burden, circulating RNA offers functional insight into transcriptional activity, cellular states, and systemic host responses that are not readily inferred from DNA alone.

By combining these modalities, multi-modal liquid biopsy frameworks may improve biological interpretability and clinical robustness. For instance, a decline in ctDNA mutation burden accompanied by concurrent activation of immune-related RNA signatures may provide complementary evidence of therapeutic response during immune checkpoint blockade [38,66]. Conversely, persistence of oncogenic mutations detected by ctDNA together with circulating RNA signatures indicative of epithelial–mesenchymal transition or metabolic reprogramming may signal early adaptive resistance [6,16]. These examples illustrate how genomic and transcriptomic layers can anchor and contextualize each other, enabling more nuanced biological inference than either modality alone [9,13]. Genomic alterations detected by ctDNA can anchor transcriptional signals to tumor origin, while RNA-based readouts can contextualize these alterations within dynamic biological processes such as immune activation, stress responses, or treatment-induced adaptation [13,15,31]. Importantly, integration may also mitigate limitations inherent to individual assays by reducing false-positive signals and enhancing signal stability across heterogeneous patient populations. Rather than replacing existing liquid biopsy platforms, circulating RNA is therefore likely to function as a complementary layer within integrated diagnostic and monitoring strategies.

### 10.2. AI-Driven Transcriptomic Signatures

The analytical complexity of circulating RNA data, characterized by high dimensionality, biological heterogeneity, and context-dependent signal patterns, positions artificial intelligence and deep learning approaches as central tools for future development [13,45,74]. Unlike traditional univariate or rule-based methods, machine learning models can capture complex, non-linear relationships across large transcriptomic feature spaces, enabling the identification of composite RNA signatures associated with disease presence, progression, or therapeutic response.

In the context of circulating RNA, AI-driven models offer particular advantages by accommodating variability in RNA abundance, carrier composition, and technical noise while extracting clinically relevant patterns [15,58]. These approaches are especially well suited for integrating transcriptomic data with genomic, proteomic, and clinical variables, thereby supporting the development of multi-modal predictive frameworks. However, the successful clinical deployment of such models will depend on transparent model construction, rigorous external validation, and careful consideration of interpretability to ensure that algorithmic predictions can be meaningfully translated into clinical decision-making.

### 10.3. Toward Functional Precision Oncology

Ultimately, the integration of circulating RNA into liquid biopsy platforms supports a broader shift toward functionally informed precision oncology. In this paradigm, treatment decisions are guided not only by static genomic alterations but also by dynamic transcriptional states, pathway activity, and host–tumor interactions that evolve over time [4,9,13]. Circulating RNA provides a unique window into these processes, offering the potential to capture real-time biological responses to therapy and disease progression.

Realizing this vision will require continued methodological innovation, including improved standardization of analytical workflows, large-scale prospective validation, and close alignment with clinical care pathways [9,15,16]. Equally important will be the development of clinically interpretable frameworks that link circulating RNA signatures to actionable therapeutic strategies. As liquid biopsy technologies mature beyond mutation-centric approaches, circulating RNA is poised to become a key component of integrated platforms designed to support adaptive, biologically informed cancer management.

## 11. Conclusions

Circulating RNA represents a biologically rich yet analytically challenging dimension of liquid biopsy that complements mutation-centric approaches based on circulating DNA. This review is intended for researchers and clinician–scientists seeking a systems-level framework that bridges molecular liquid biopsy technologies with functionally informed oncology applications. By reflecting active transcriptional programs, cellular states, and systemic host responses, circulating RNA provides access to functional information that is often invisible to genomic analyses alone.

Throughout this review, circulating RNA was conceptualized as a *liquid transcriptome*, emphasizing its value as an integrated readout of tumor-intrinsic activity, host immune and inflammatory responses, and intercellular communication. Importantly, the clinical relevance of circulating RNA emerges not from isolated biomarkers, but from integrative interpretation across physical carriers, RNA biotypes, and biological meaning.

Although substantial challenges remain—including pre-analytical variability, analytical instability, and computational complexity—ongoing methodological advances and an expanding body of clinical evidence suggest that these barriers are increasingly tractable. In particular, integration with other liquid biopsy modalities and adoption of transparent, externally validated analytical frameworks are expected to accelerate clinical translation.

As liquid biopsy strategies continue to evolve beyond static genomic profiling, circulating RNA is well positioned to play a central role in enabling functionally informed precision oncology, where clinical decisions are guided by dynamic biological states rather than genetic alterations alone.

## Figures and Tables

**Table 1:** Physical carriers of circulating RNA and their analytical characteristics.

Physical Carrier	Stability in Circulation	Biological Selectivity	Protection from Degradation	Implications for Analytical Workflows
**Cell-free RNA**	Low	Low (largely stochastic release)	Minimal; highly susceptible to RNases	Requires rapid processing and stringent pre-analytical control; limited reproducibility
**Extracellular vesicle-associated RNA**	High	High (active, cargo-selective packaging)	Strong protection by lipid bilayer	Requires EV isolation and characterization; increased complexity and cost but higher biological specificity
**Ribonucleoprotein-associated RNA**	Moderate to high	Moderate (protein-mediated stabilization)	Protected by RNA-binding proteins	Sensitive to extraction protocols; intermediate analytical complexity

**Table 2:** Major RNA biotypes detected in circulation and their biological and analytical features. Summary of circulating RNA biotypes, including mRNA, miRNA, lncRNA, and circRNA, highlighting their stability, biological roles, cancer relevance, and key analytical considerations.

RNA Biotype	Typical Carrier(s)	Biological Role	Cancer Relevance	Analytical Considerations
mRNA	cfRNA, EV-RNA	Encodes protein-coding transcripts reflecting active gene expression	Tissue-of-origin inference, pathway activity, treatment response	Fragmented, low abundance; requires sensitive library preparation and normalization
miRNA	RNP-RNA, EV-RNA	Post-transcriptional gene regulation	Oncogenic and tumor-suppressive regulation; widely studied biomarkers	High stability; risk of low specificity when used individually
lncRNA	cfRNA, EV-RNA	Epigenetic and transcriptional regulation	Cancer-type and state specificity	Low abundance; incomplete annotation; higher noise
circRNA	EV-RNA, cfRNA	Regulatory RNA with circular structure	Emerging cancer biomarkers with high stability	Detection requires junction-aware algorithms; limited functional annotation

mRNA, messenger RNA; miRNA, microRNA; lncRNA, long non-coding RNA; circRNA, circular RNA; cfRNA, cell-free RNA; EV, extracellular vesicle; RNP, ribonucleoprotein.

**Table 3:** Biological interpretation layers of circulating RNA signals in cancer. Overview of tissue-of-origin, cell-state-associated, and host-response transcriptomic signals, with representative examples and potential clinical applications in cancer diagnosis and treatment.

Interpretation Layer	Representative Transcriptomic Features	Biological Meaning	Potential Clinical Applications	Representative Clinical Context
**Tissue-of-origin signals**	Organ-enriched mRNAs, tissue-specific lncRNAs	Reflect the tissue or organ source of circulating RNA signals	Multi-cancer detection, primary site inference	Cancer of unknown primary (CUP), multi-cancer early detection
**Cell-state signals**	Proliferation signatures, hypoxia-related genes, EMT programs, metabolic pathway transcripts	Indicate functional states and adaptive responses of tumor cells	Early detection, monitoring of treatment response and resistance	Early therapeutic escape, adaptive resistance before radiographic progression
**Host-response signals**	Immune activation markers, interferon signaling, inflammatory transcripts	Capture systemic immune and inflammatory responses to cancer	Immunotherapy stratification, response and toxicity monitoring	Immune checkpoint inhibitor response, immune-related adverse events

lncRNA, long non-coding RNA; EMT, epithelial–mesenchymal transition.

**Table 4:** Representative clinical and translational studies utilizing circulating RNA in cancer liquid biopsy. The listed studies are not intended to provide an exhaustive catalog of all clinical trials but rather to illustrate the current scope, clinical contexts, and emerging applications of circulating RNA across cancer types.

Cancer Type	Study Type	Sample Source	RNA Analyte	Clinical Application	Comparator	Cohort (*n*)	Key Finding (Summary)	Ref.
Multiple solid tumors	Prospective, translational	Plasma	cfRNA (transcriptome)	Cancer detection	Healthy controls	283	Plasma cfRNA signatures distinguish cancer vs. non-cancer	[63]
Hepato-cellular carcinoma	Prospective	Plasma + single-cell	cfRNA	Tumor detection/characterization	Tissue biopsy	41	cfRNA captures tumor-associated transcriptional programs	[61]
Pancreatic cancer	Case–control	Plasma	cfRNA	Early detection	Imaging, controls	94	cfRNA signatures detect cancer in high-risk and symptomatic cohorts	[56]
DLBCL/PMBCL	Explora-tory	Plasma	Total cfRNA	Disease characterization	Clinical subtype	54	cfRNA profiles reflect lymphoma subtype biology	[62]
Melanoma (IO)	Prospective clinical	Whole blood	Immune mRNA	Response prediction	RECIST	210	IFN-γ-related RNA signature predicts PD-1 response	[38]
Melanoma (IO)	Prospective clinical	Whole blood	Immune gene signature	Toxicity prediction	irAE outcomes	109	RNA signature associated with CTLA-4-related diarrhea	[69]
Advanced solid tumors	Longitudinal clinical	Plasma + ctDNA	cfRNA + ctDNA	Treatment monitoring	ctDNA alone	89	Combined RNA–DNA profiling improves clinical association	[70]
Colorectal cancer	Translational	Serum EVs	circRNA	Chemo-resistance	Treatment response	60	EV-derived circRNAs associated with chemo-resistance	[80]

cfRNA, cell-free RNA; ctDNA, circulating tumor DNA; IO, immune-oncology; RECIST, Response Evaluation Criteria in Solid Tumors; irAE, immune-related adverse event; IFN-γ, interferon-gamma; PD-1, programmed cell death protein 1; CTLA-4, cytotoxic T-lymphocyte-associated protein 4; EV, extracellular vesicle.

## Data Availability

No new data were created or analyzed in this study. Data sharing is not applicable to this article.
